# Glucocerebrosidase mutations and Parkinson disease

**DOI:** 10.1007/s00702-022-02531-3

**Published:** 2022-08-06

**Authors:** Sophia R. L. Vieira, Anthony H. V. Schapira

**Affiliations:** Department of Clinical and Movement Neurosciences, University College London Queen Square Institute of Neurology, Rowland Hill St., London, NW3 2PF UK

**Keywords:** GBA1, Parkinson disease, Neurodegeneration, Genetics, Gaucher disease, Ambroxol

## Abstract

The discovery of glucocerebrosidase (GBA1) mutations as the greatest numerical genetic risk factor for the development of Parkinson disease (PD) resulted in a paradigm shift within the research landscape. Efforts to elucidate the mechanisms behind GBA1-associated PD have highlighted shared pathways in idiopathic PD including the loss and gain-of-function hypotheses, endoplasmic reticulum stress, lipid metabolism, neuroinflammation, mitochondrial dysfunction and altered autophagy–lysosomal pathway responsible for degradation of aggregated and misfolded a-synuclein. GBA1-associated PD exhibits subtle differences in phenotype and disease progression compared to idiopathic counterparts notably an earlier age of onset, faster motor decline and greater frequency of non-motor symptoms (which also constitute a significant aspect of the prodromal phase of the disease). GBA1-targeted therapies have been developed and are being investigated in clinical trials. The most notable are Ambroxol, a small molecule chaperone, and Venglustat, a blood–brain-barrier-penetrant substrate reduction therapy agent. It is imperative that further studies clarify the aetiology of GBA1-associated PD, enabling the development of a greater abundance of targeted therapies in this new era of precision medicine.

## Introduction

Biallelic (homozygous or compound heterozygous) mutations in the *GBA1* gene, encoding the lysosomal enzyme glucocerebrosidase (GCase; EC 3.2.1.45), are pathognomonic for the commonest lysosomal storage disorder (LSD) Gaucher disease (GD) (Grabowski 2008). Heterozygous *GBA1* variants account for the most significant genetic risk factor for Parkinson disease (PD), the second most common neurodegenerative disease following Alzheimer’s disease with a reported prevalence rate of 315 per 100,000 persons worldwide (Sidransky et al. 2009; Pringsheim et al. 2014). Approximately, 5–30% of PD patients carry *GBA1* mutations; variations in the prevalence rate of *GBA1* mutations can be attributed to the population studied and extent of exome sequencing (Sidransky et al. 2009; Duran et al. 2013; Schapira 2015; Cilia et al. 2016, Senkevich and Gan-Or 2020). Clinically, *GBA1*-associated PD (*GBA1*-PD) mirrors idiopathic PD (iPD), albeit significant differences include an earlier age of disease onset, greater frequency of non-motor symptoms (NMSs) and cognitive impairment (Petrucci et al. 2020; Avenali et al. 2021). *GBA1* mutation-positive individuals exhibit an increased risk of developing Dementia with Lewy Bodies (DLB; odds ratio, OR ~ 8), notably higher than that for PD (OR 3.5–6) (Neumann et al. 2009; Sidransky et al. 2009; McKeith et al. 2017). In this review, we provide an update on the association between *GBA1* mutations and PD and how future neuroprotective therapies may target the *GBA1* pathway.

## *GBA1* mutations and Gaucher disease

Biallelic *GBA1* mutations cause GD, the commonest LSD, characterised by GCase deficiency. The enzyme GCase is involved in glycosphingolipid and ganglioside metabolism, cleaving glucosylceramide (GlcCer) and glucosylsphingosine (GlcSph) into ceramide and glucose, and sphingosine and glucose, respectively (Brady et al. 1965). Reduced GCase activity leads to GlcCer and GlcSph accumulation in the Gaucher cells (lysosomes of macrophages) within the liver, bone marrow and spleen (Rosenbloom and Weinreb 2013). GD is particularly prevalent in the Ashkenazi Jewish (AJ) population (118 per 100,000) compared to non-AJ populations (1–2 per 100,000) (Mistry et al. 2011). GD presents with variable symptomatology; the highly heterogeneous disease can be stratified into three types according to severity and neurological involvement. Type I (non-neuronopathic) GD accounts for 95% of cases with a spectrum of clinical presentations from subclinical patients to hallmark symptoms of hepatosplenomegaly, pancytopenia and osteoporosis (Grabowski et al. 2015). Neuronopathic GD is categorised into patient subgroups demonstrating rapid (type 2 GD) and slower (type 3 GD) neurological deterioration (Mistry et al. 2015). Neurological symptoms include myoclonic or generalised seizures and eye movement abnormalities (saccades and supranuclear ophthalmoplegia) (Mistry et al. 2015). Different *GBA1* mutations are more represented in specific phenotypes of GD. The ‘mild’ GD type 1 is found with the N370S *GBA1* mutation and a proportion of homozygous N370S *GBA1* mutation-positive patients remain asymptomatic (Hruska et al. 2008). The L444P *GBA1* mutation has been reported in patients exhibiting severe neurological symptoms, accounting for over 40% of mutations in neuronopathic forms of GD (Koprivica et al. 2000; Stone et al. 2000). Such ‘mild’ and ‘severe’ *GBA1* mutations are associated with an in vitro residual GCase enzymatic activity of 32–38% and 13–24%, respectively (Alfonso et al. 2004; Sidransky and Lopez 2012; Malini et al. 2014). Others have failed to replicate such findings, instead reporting an overlap in the range of GCase enzymatic activity for severe and mild *GBA1* mutations (Sidransky 2004). Analyses of GCase activity within the lysosome only may offer more accurate and consistent readings. The wide spectrum of clinical manifestations of GD hinders a clear-cut classification of GD in practice, particularly for neuronopathic forms of GD (Goker-Alpan et al. 2003). Mounting evidence supports the notion that GD is likely to be a spectrum disorder instead (Beavan et al. 2015). Current treatments for GD aim to enhance GCase activity via enzyme replacement therapy (ERT) and substrate reduction therapy (SRT). Newer techniques, such as, chaperone molecules are under current investigation (Parenti 2009). Administered intravenously, ERT supplies recombinant GCase enzymes, modified to facilitate efficient uptake via macrophages. ERT is a treatment option in type 1 GD, but since ERT cannot penetrate the blood–brain barrier (BBB), its potential use in ameliorating neurological involvement in GD is limited (Valayannopoulos 2013; Jung et al. 2016). A more upstream therapy, SRT, utilises GlcCer synthase inhibitors to reduce GlcCer accumulation (Lukina et al. 2019).

The *GBA1* gene localises to chromosome 1q21, comprising 7 kb with 11 exons and 10 introns. A highly homologous pseudogene, *GBA1P* (with 96% exonic sequence homology), is located 12 kb downstream, enabling recombination events between *GBA1P* and *GBA1* which generate multiple complex alleles (Hruska et al. 2008). Over 300 *GBA1* mutations have been reported; more than 100 *GBA1* variants, not termed mutations as they are not pathogenic for GD, are associated with PD (Guo et al. 2015; Fan et al. 2016; O'Regan et al. 2017). 90% of *GBA1* mutations in AJ patients are N370S (or N409S according to the latest nomenclature), L444P (L483P), IVS2 + 1G > A or 84insG (Gomez et al. 2017). Interestingly, *GBA1* mutations exhibit ethnic heterogeneity (Zhang et al. 2018a). N370S and L444P *GBA1* mutations are frequently detected in AJ GD patients and non-AJ Caucasians, respectively (Grabowski and Horowitz 1997; Hruska et al. 2008). Asian ethnic groups frequently harbour L444P and F252I *GBA1* mutations in neuronopathic GD subtypes, with N370S rarely found (Hruska et al. 2008; Riboldi and Di Fonzo 2019). Over the last two decades, an association between GD and PD has been noticed, initially through case reports of parkinsonian symptoms among GD patients (Tayebi et al. 2003; Aharon-Peretz et al. 2004; Beavan et al. 2015) and confirmed by a large multicentre study (Sidransky et al. 2009).

The mature GCase protein is a 497-amino-acid protein assembled on endoplasmic reticulum (ER) and trafficked to the lysosome by binding to the LIMP2 receptor where it is activated by its cofactor, Saposin C (Grabowski et al. 1990). Three structural domains of GCase have been identified using X-crystallography: domain I contains an antiparallel beta-sheet and structural loop (residues 1–27 and 383–414); domain II made up of eight beta-sheets, resembling an immunoglobulin fold (residues 30–75 and 431–497) and domain III harbours the active site within a (β/α)_8_ triophosphate isomerase (TIM) barrel (residues 76–381 and 416–430) (Kacher et al. 2008).

## *GBA1* mutations and Parkinson disease

PD is a common neurodegenerative disorder characterised by a progressive loss of dopaminergic neurones in the substantia nigra pars compacta (SNpc) and intracytoplasmic inclusions predominantly composed of aggregated a-synuclein (Lewy bodies) (Pellicano et al. 2007). PD is clinically defined by the presence of the cardinal motor symptoms of rigidity, bradykinesia and tremor (Postuma et al. 2015). NMSs, such as neuropsychiatric symptoms, autonomic dysfunction, hyposmia, rapid eye movement sleep disorder (RBD) and cognitive impairment, are a prominent feature of PD but remain largely resistant to dopaminergic therapy given the involvement of other neurotransmitters (Schapira et al. 2017). Recent studies have highlighted a prodromal phase, characterised by NMSs and mild motor signs, which may precede clinical diagnosis of PD by up to 20 years (Gustafsson et al. 2015; Schrag et al. 2015; Fereshtehnejad et al. 2019). Accurate identification of the prodromal phase will be critical for the efficacy of potential neuroprotective therapies given that 50–70% of SNpc dopaminergic neurones have already undergone degeneration prior to the onset of motor symptoms (Dauer and Przedborski 2003; Ross et al. 2004).

The association between *GBA1* mutations and PD was first observed in clinic over 20 years ago following reports of parkinsonian features in GD patients and *GBA1* mutation carriers (Neudorfer et al. 1996; Machaczka et al. 1999). Tayebi et al. (2003) revealed the presence of non-neuronopathic N370S *GBA1* mutations in GD patients with Parkinsonism. Autopsy studies of GD brains added further support to the GD–PD association through findings of a significant loss of substantia nigra dopaminergic neurones alongside extensive cytotoxic Lewy body aggregation (Tayebi et al. 2003; Wong et al. 2004). Confirmation of this genetic link was later provided by a large multicentre study of 5691 PD patients and 4898 controls, with an OR of 5.43 for *GBA1* (Sidransky et al. 2009). Subsequent genetic analyses replicated such results, demonstrating that *GBA1* mutations represent the greatest numerical genetic risk factor for PD (Chen et al. 2014; Nalls et al. 2014; Robak et al. 2017). Both homozygous and heterozygous *GBA1* mutations have similar ORs for PD (Alcalay et al. 2014). Penetrance of *GBA1* mutations is variable and age dependent; a cumulative risk of developing PD of 5% and 10–30% is observed by 60 and 80 years of age, respectively, in heterozygous carriers when compared to controls (Anheim et al. 2012; Rana et al. 2013; Balestrino et al. 2020). Causes underlying the relatively low rate of PD phenoconversion in individuals with biallelic or heterozygous GBA1 mutations remain elusive (Anheim et al. 2012). Ethnicity also influences the extent by which *GBA1* mutations result in an increased PD risk (Zhang et al. 2018a). PD risk is associated with the following *GBA1* mutations: R496H and 84insGG in AJ populations; L444P, R120W, IVS2 + 1G > A, H255Q, D409H, RecNciI, E326K, T369M in non-AJ populations; N370S, H255Q, D409H, E326K in European/West Asians; R120W in East Asians, while N370S conveys a pan-ethnic PD risk (Zhang et al. 2018a).

## Features of *GBA1*-associated Parkinson disease

### Clinical features

Initial studies reported no significant differences in clinical trajectories between iPD and *GBA1*-PD (Aharon-Peretz et al. 2005). Key subtle traits of *GBA1*-PD have since emerged notably, an earlier age of onset by ~ 1.7–6.0 years, higher Unified Parkinson’s Disease Rating Scale Part III (UPDRS-III) scores, greater frequency of dementia, visual hallucinations and severity of NMSs, particularly depression (Neumann et al. 2009; Hu et al. 2010; Winder-Rhodes et al. 2013; Asselta et al. 2014; Zhang et al. 2018b). Notably, there is no apparent correlation between PD clinical phenotype or severity and GCase activity (Alcalay et al. 2020; Omer et al. 2022), although cognitive dysfunction may be an exception to this (see below).

Clinically, *GBA1*-PD patients exhibit motor symptoms of tremor, rigidity, and bradykinesia, with the latter more commonly observed in the earliest phases of disease in this cohort (Ziegler et al. 2007; Lesage et al. 2011). A faster progression and deterioration in disease course in *GBA1*-PD patients has been reported extensively in literature, concomitant with a reduced survival rate, compared to iPD (Winder-Rhodes et al. 2013; Brockmann et al. 2015). Interestingly, non-pathogenic *GBA1* variants may indeed affect motor symptomatology, with polymorphisms also associated with a greater risk of motor deterioration (Winder-Rhodes et al. 2013; Jesus et al. 2016). Motor complications, wearing off, delayed on and dyskinesias, are more prevalent in *GBA1*-PD, especially in those harbouring severe mutations, and occur earlier than in *GBA1* mutation-negative PD patients (Jesus et al. 2016; Zhang et al. 2018b).

NMSs form a significant aspect of the *GBA1*-PD disease course. PD patients with *GBA1* mutations exhibit a threefold increased risk of cognitive decline, affecting working memory, executive and visuospatial functions (Alcalay et al. 2012; Zokaei et al. 2014; Mata et al. 2016; Leocadi et al. 2022). The degree of cognitive impairment is correlated with the severity of *GBA1* mutations (Petrucci et al. 2020; Szwedo et al. 2022). Deficient clearance mechanisms of a-synuclein in *GBA1* mutants leading to accelerated a-synuclein pathology in cortical areas is thought to underlie the increased rates of cognitive impairment and ultimately, dementia in *GBA1*-PD patients (Jesus et al. 2016). Partial support exists for a subtle alteration in cognitive functioning in *GBA1* mutation-positive individuals without PD (Avenali et al. 2019; Moran et al. 2021). Ongoing longitudinal studies aim to identify cognitive decline, other NMS and motor abnormalities in individuals harbouring *GBA1* mutations prior to the onset of PD symptoms (Higgins et al. 2021). Data suggest that GBA1-PD has a stronger association with depression than iPD (Brockmann et al. 2011; Swan et al. 2016), albeit conflicting findings have been obtained (Zhang et al. 2015). Anxiety risk has been less extensively investigated in *GBA1*-PD, with only partial evidence supporting an increased risk in this patient cohort (Wang et al. 2014). Further, autonomic symptoms have been frequently reported in *GBA1*-PD patients including hyposmia, constipation, orthostatic hypotension and urogenital dysfunction (Brockmann et al. 2011).

### Imaging

It is not currently possible to differentiate *GBA1*-PD from iPD based on neuroimaging alone (Barrett et al. 2013). Non-manifesting *GBA1* mutation carrier status is not associated with reduced striatal dopaminergic tone (Goker-Alpan et al. 2012; Lopez et al. 2020; Simuni et al. 2020; Mullin et al. 2021). Utilising fluorodopa PET and transcranial sonography, no significant difference in nigrostriatal imaging has been reported between iPD and *GBA1*-PD patients (Kono et al. 2007; Kraoua et al. 2009; Goker-Alpan et al. 2012; Barrett et al. 2013; Lopez et al. 2020). Longitudinal investigations using single-photon emission computerised tomography (SPECT) and structural MRI have revealed a more accelerated disease course in *GBA1*-PD compared to iPD (Caminiti et al. 2022; Leocadi et al. 2022). Of note, iPD patients demonstrated similar patterns of cortical thinning to GBA1-PD 5 years post baseline MRI scans (Leocadi et al. 2022). The finding of a more aggressive progression in *GBA1*-PD cases has also been supported by cerebral blood flow (Goker-Alpan et al. 2012; Cilia et al. 2016) and FDG-PET studies (Greuel et al. 2020; Schindlbeck et al. 2020). Imaging in GBA1-PD has recently been reviewed (Filippi et al. 2022).

### Neuropathology

Neuropathology studies of *GBA1*-PD brains have reported Lewy body aggregates in cortical regions, notably including the hippocampal regions CA2–4, in addition to the substantia nigra which is classically affected in iPD (Wong et al. 2004; Clark et al. 2009). Of note, others have not replicated such results (Parkkinen et al. 2011). Further reports indicate an increased rate of co-aggregation of GCase and a-synuclein in Lewy bodies in *GBA1*-PD brains (Goker-Alpan et al. 2010). Enhanced microglial activation in *GBA1* mutation carriers without PD or dopaminergic loss has recently been reported (Mullin et al. 2021).

## Mechanisms of GBA1-associated Parkinson disease

GCase activity is reduced to 58%, 67% and < 15% of normal function in *GBA1*-PD, iPD and GD patients, respectively (Gegg et al. 2012, Sidransky 2012). A minority of GD patients develop PD, indicating that PD risk is not proportional to GCase activity (Sidransky 2012). Numerous research efforts are underway to elicit pathways which may interact with GCase to increase PD risk in *GBA1* mutation carriers, and whether such pathways are relevant to iPD patients. Proposed mechanisms include ER stress, autophagic lysosomal dysfunction, abnormal lipid homeostasis, mitochondrial dysfunction and neuroinflammation (Smith and Schapira 2022).

*GBA1* gain-of-function mutations may lead to misfolded GCase being sequestered in the ER, impairing ER-associated degradation (ERAD) and triggering ER stress (Fernandes et al. 2016)). A-synuclein accumulation in the ER further impedes ER–Golgi transport (Cooper et al. 2006), upregulating the unfolded protein response (UPR) (Maor et al. 2013; Sanchez-Martinez et al. 2016). Induced pluripotent stem cell (iPSC)-derived neurones harbouring *GBA1* mutations showed an increase in a-synuclein release following the activation of ER stress (Schondorf et al. 2014). Interestingly, GCase inhibitor, conduritol B epoxide (CBE), also produced cellular changes indicative of ER stress, providing support for both the loss and gain-of-function hypotheses in *GBA1*-PD pathogenesis (Kurzawa-Akanbi et al. 2012). Further, evaluations of *GBA1*-PD post-mortem brains have revealed altered levels of UPR-associated proteins, BiP, CHOP and HERP (Gegg et al. 2012; Kurzawa-Akanbi et al. 2012). GCase chaperone, Ambroxol, reversed the activation of UPR and was subsequently investigated as a disease-modifying therapy for PD in clinical trials (Suzuki et al. 2015b; Maor et al. 2016; Sanchez-Martinez et al. 2016). Moreover, it is noteworthy that ER stress may be an important therapeutic target in several neurodegenerative diseases (Kanekura et al. 2006; Yang et al. 2010; Liu et al. 2013).

GCase is involved in lysosomal clearance of a-synuclein (Mazzulli et al. 2011). GCase knockdown compromised lysosomal degradation of proteins, resulting in a 40% reduced rate of proteolysis (Mazzulli et al. 2011). Lysosomal/autophagic dysfunction contributes to the development of PD in *GBA1* mutation carriers (Mazzulli et al. 2011; Magalhaes et al. 2016). Accumulation of a-synuclein and alterations in autophagy–lysosomal pathways occur concurrently in GCase deficient models (Cullen et al. 2011; Chiasserini et al. 2015; Rocha et al. 2015b). Autophagic impairment has also been noted in iPD brains (Alvarez-Erviti et al. 2010). A recent study reported that 50% of GCase in GBA-PD brain was present on the lysosomal surface and impaired chaperone-mediated autophagy, thereby increasing α-synuclein levels (Kuo et al. 2022).

GlcCer and GlcSph accumulation has been found in various GCase deficient models (Sardi et al. 2011; Xu et al. 2011; Farfel-Becker et al. 2014; Srikanth et al. 2021; Galvagnion et al. 2022). Serum ceramide, monohexosylceramide and sphingomyelin levels were raised in *GBA1*-PD patients compared to iPD (Guedes et al. 2017). GlcCer promotes the formation of proteinase K-resistant a-synuclein, suggesting it is involved in aggregating misfolded a-synuclein (Suzuki et al. 2015a). Lipids from *GBA1*-L444P fibroblasts accelerated a-synuclein aggregation compared to controls, with a-synuclein–lipid co-assembly indicating an active role for lipids in a-synuclein pathology (Galvagnion et al. 2022). Indeed, increased GlcSph levels are associated with increased substantia nigra p129/total α-synuclein ratios (Gundner et al. 2019). Ser129 phosphorylation of a-synuclein is present in > 90% of Lewy bodies, correlating with the progression of PD pathology (Fujiwara et al. 2002; Lue et al. 2012). Further, a link between lipid accumulation and worsening cognition in iPD patients has been suggested (Mielke et al. 2013). Normalising lipid levels in a *GBA1*-PD mouse model improved cognitive deficits (Sardi et al. 2017). However, whilst GlcCer and GlcSph accumulation has been observed in iPD and GD brains (Orvisky et al. 2002; Huebecker et al. 2020), it remains absent from the putamen and cerebellum of *GBA1*-PD patients (Gegg et al. 2012).

Mitochondrial dysfunction and oxidative stress play a key role in PD (Schapira et al. 1989). Reports of impaired mitochondrial function and morphology have emerged in numerous GCase deficient animal and cell models (Osellame et al. 2013; Garcia-Sanz et al. 2017; Li et al. 2019). Post CBE exposure, SHSY-5Y cells showed a progressive decline in mitochondrial membrane potential, increased free radical generation and a-synuclein levels (Cleeter et al. 2013). Similar findings were found in iPSC-derived neurones from *GBA1*-PD patients, albeit no gene dosage effect on mitochondrial function was observed between *GBA1* heterozygous and knockout neurones (Schondorf et al. 2018). Different mechanisms may underlie the abnormal mitochondrial function according to *GBA1* mutation number (Schondorf et al. 2018). Dysfunctional autophagy–lysosomal degradation pathways may contribute to the abnormal mitochondrial function observed in GCase deficient models (Schondorf et al. 2018). Further, L444P *GBA1* knock-in mice displayed mitochondrial defects, with the concomitant a-synuclein accumulation in dopaminergic neurones rendering them more susceptible to the neurotoxin, MPTP (Yun et al. 2018).

Mounting evidence implicates neuroinflammation in PD pathogenesis, with elevated immune markers reported in the plasma (Miliukhina et al. 2020) and brain tissue (Mullin et al. 2021) isolated from *GBA1*-PD patients. Upregulation of complement C1q has been noted in the brains of mice following CBE exposure (Rocha et al. 2015b). GCase deficiency may increase the sensitivity of microglia to various insults, leading to an increased risk of neurodegeneration (Brunialti et al. 2021). Moreover, the finding of an association between HLA alleles and an increased risk of PD adds further support to the role of neuroinflammation in the disease process (International Parkinson’s Disease Genomics and Wellcome Trust Case Control 2011).

## Potential *GBA1*-targeting therapies for PD

### Enzyme replacement therapy and substrate reduction therapy

ERT and SRT has FDA approval for the treatment of GD. Preclinical work reported diminished a-synuclein pathology in human midbrain dopamine neurones post SRT administration (Zunke et al. 2018). However, the inability of such therapies to cross the BBB has limited its potential use. Alternative delivery methods to enhance neural GCase are under investigation (Poewe et al. 2017). Methods including BBB-penetrant peptides, exosome-mediated delivery and transport vehicle modified recombinant GCase may improve delivery of ERT (Gramlich et al. 2016; Hall et al. 2016; Ysselstein et al. 2021). Experimental BBB-penetrating SRT Venglustat was safe, well tolerated and demonstrated sufficient target engagement with a reduction in a-synuclein levels reported in the Phase I MOVES-PD clinical trial (Peterschmitt et al. 2022). A recent Phase II study of Venglustat revealed no such clinical benefit, with poorer performance in motor function observed following administration in a *GBA1*-PD cohort (Peterschmitt et al. 2021). However, it is known that specific *GBA1* mutations affect the GCase protein differently, perhaps some therapies may be more beneficial when stratifying mutation carriers (Gan-Or et al. 2018).

### Gene therapy

Replacement of mutant *GBA1* gene via the adeno-associated virus (AAV) may be a therapeutic option in *GBA1*-PD (Hudry and Vandenberghe 2019). AAVs do not integrate into host genomes and possess a strong safety profile (Hudry and Vandenberghe 2019). AAV-GBA1 delivery in animal models has been reported to enhance GCase levels, reduce neuroinflammation and the accumulation of GlcSph, a-synuclein, ubiquitin and tau (Sardi et al. 2013; Rocha et al. 2015a; Massaro et al. 2018; Sucunza et al. 2021). Improved motor and memory function were also observed in mice and macaques following AAV-*GBA1* administration (Sardi et al. 2013; Massaro et al. 2018). A Phase I/IIa clinical trial is currently underway to ascertain the safety, tolerability and clinical efficacy of AAV-*GBA1* vector PR001 (LY3884961) in GBA1-PD patients and children with type 2 GD (ClinicalTrials.gov Identifier: NCT04127578 and NCT04411654, respectively).

### Molecular chaperones

**Fig. 1:**
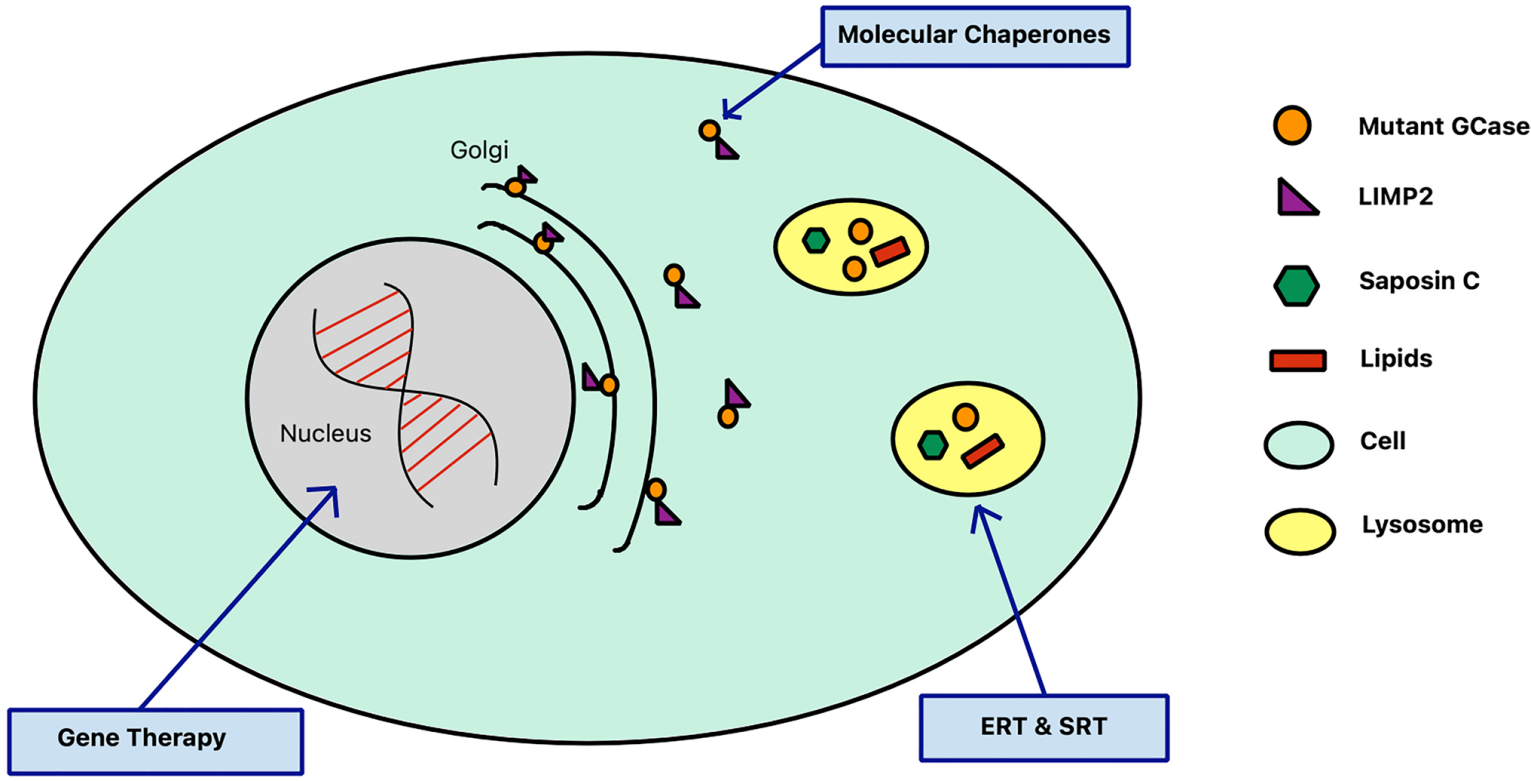
The various approaches currently being explored in the search for neuroprotective therapies in *GBA1*-PD. Gene therapy may be used to replace the faulty *GBA1* gene. Molecular chaperones may increase GCase activity by structurally correcting misfolded GCase. The use of enzyme replacement therapy (ERT) and substrate reduction therapy (SRT) to replenish GCase levels and reduce GlcCer accumulation in *GBA1*-PD, respectively, is being investigated

Chaperone therapy to disentangle misfolded GCase in ER and stabilise for transport to the lysosome is a promising therapeutic avenue in *GBA1*-PD (Fig. 1) (Ambrosi et al. 2015). The mucolytic ambroxol is a small molecule inhibitory chaperone capable of increasing GCase activity in mice, non-human primates and *GBA1*-PD patient cells (McNeill et al. 2014; Migdalska-Richards et al. 2016, 2017). Clinical trials are currently evaluating the efficacy of oral ambroxol in type 1 GD, PD dementia and *GBA1*-PD (ClinicalTrials.gov Identifier: NCT03950050, NCT02914366 and NCT02941822, respectively). Interestingly, ambroxol therapy achieved sufficient CSF penetration and target engagement, augmenting GCase protein levels (Mullin et al. 2020). Non-inhibitory chaperones, such as LTI-291, do not interact with an enzyme’s active site, and have been found to increase GCase activity and half-life in a phase I clinical trial (Trialregister.nl ID: NTR7299) (den Heijer et al. 2021).

## Conclusion

Significant insights into the pathogenesis of PD have been obtained following the finding of GBA1 mutations as the greatest numerical risk factor for PD. This link highlighted ER stress, lipid metabolism and autophagy–lysosomal pathways as key disease-causing mechanisms. *GBA1*-targeted therapies have been developed, with their clinical efficacy being evaluated in trials. It remains imperative to understand why only a minority of *GBA1* mutation carriers progress to PD phenoconversion. Understanding more about the *GBA1*-PD link may help elucidate better treatments and methods of predicting who will develop the disease prior to symptom onset.
